# Poly[diammonium [(μ_4_-butane-1,2,3,4-tetra­carboxyl­ato)zincate] tetra­hydrate]

**DOI:** 10.1107/S1600536812038883

**Published:** 2012-09-15

**Authors:** Shouwen Jin, Yanfei Huang, Shuaishuai Wei, Yong Zhou, Yingping Zhou

**Affiliations:** a Tianmu College of ZheJiang A & F University, Lin’An 311300, People’s Republic of China

## Abstract

In the title compound, {(NH_4_)_2_[Zn(C_8_H_6_O_8_)]·4H_2_O}_*n*_, the asymmetric unit contains one ammonium cation, half of a butane-1,2,3,4-tetra­carboxyl­ate anion, one Zn^2+^ cation and two water mol­ecules. The butane-1,2,3,4-tetra­carboxyl­ate ligand is located about an inversion centre at the mid-point of the central C—C bond. The Zn^2+^ cation is situated on a twofold rotation axis and is surrounded by four O atoms from four symmetry-related butane-1,2,3,4-tetra­carboxyl­ate anions in a distorted tetra­hedral environment. In turn, each anion coordinates to four Zn^2+^ cations. The bridging mode of the anions leads to a three-dimensional framework structure with channels extending along [110] and [010] in which the ammonium cations and the water mol­ecules are located. N—H⋯O and O—H⋯O hydrogen bonding between the cations and water mol­ecules and the uncoordinating O atoms of the carboxyl­ate groups consolidates the crystal packing.

## Related literature

For general background to coordination compounds derived from carb­oxy­lic acids, see: Jin & Chen (2007*a* ▶,*b* ▶); Jin *et al.* (2007 ▶); Rueff *et al.* (2001 ▶); Strachan *et al.* (2007 ▶). For hydrogen bonding, see: Desiraju (2002 ▶).
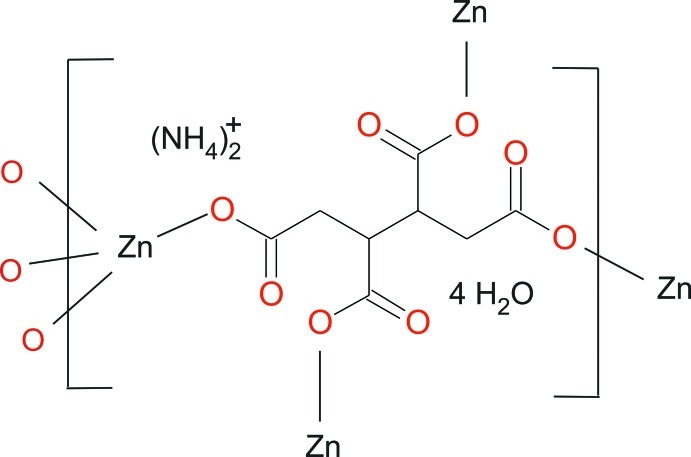


## Experimental

### 

#### Crystal data

(NH_4_)_2_[Zn(C_8_H_6_O_8_)]·4H_2_O
*M* *_r_* = 403.65
Monoclinic, 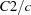

*a* = 14.1153 (12) Å
*b* = 8.8505 (8) Å
*c* = 13.5704 (11) Å
β = 111.761 (2)°
*V* = 1574.5 (2) Å^3^
*Z* = 4
Mo *K*α radiation
μ = 1.63 mm^−1^
*T* = 298 K
0.36 × 0.19 × 0.12 mm

#### Data collection

Bruker SMART 1K CCD area-detector diffractometer
Absorption correction: multi-scan (*SADABS*; Bruker, 2002 ▶) *T*_min_ = 0.697, *T*_max_ = 0.823
3841 measured reflections
1386 independent reflections
1167 reflections with *I* > 2σ(*I*)
*R*_int_ = 0.089

#### Refinement

*R*[*F* ^2^ > 2σ(*F* ^2^)] = 0.059
*wR*(*F* ^2^) = 0.156
*S* = 1.02
1386 reflections
105 parameters
H-atom parameters constrained
Δρ_max_ = 1.53 e Å^−3^
Δρ_min_ = −1.30 e Å^−3^

### 

Data collection: *SMART* (Bruker, 2002 ▶); cell refinement: *SAINT* (Bruker, 2002 ▶); data reduction: *SAINT*; program(s) used to solve structure: *SHELXS97* (Sheldrick, 2008 ▶); program(s) used to refine structure: *SHELXL97* (Sheldrick, 2008 ▶); molecular graphics: *SHELXTL* (Sheldrick, 2008 ▶); software used to prepare material for publication: *publCIF* (Westrip, 2010 ▶).

## Supplementary Material

Crystal structure: contains datablock(s) global, I. DOI: 10.1107/S1600536812038883/wm2679sup1.cif

Structure factors: contains datablock(s) I. DOI: 10.1107/S1600536812038883/wm2679Isup2.hkl

Additional supplementary materials:  crystallographic information; 3D view; checkCIF report

## Figures and Tables

**Table 1:** Hydrogen-bond geometry (Å, °)

*D*—H⋯*A*	*D*—H	H⋯*A*	*D*⋯*A*	*D*—H⋯*A*
O6—H6*D*⋯O4^i^	0.85	1.97	2.814 (4)	173
O6—H6*C*⋯O3^ii^	0.85	1.99	2.833 (5)	173
O5—H5*D*⋯O6^iii^	0.85	1.97	2.805 (5)	168
O5—H5*C*⋯O1	0.85	1.98	2.816 (4)	167
N1—H1*B*⋯O2^i^	0.90	2.31	2.981 (5)	131
N1—H1*B*⋯O3^i^	0.90	2.30	3.009 (5)	136
N1—H1*A*⋯O5^iv^	0.90	2.54	3.153 (5)	126
N1—H1*A*⋯O6^v^	0.90	2.24	2.948 (5)	135
N1—H1*D*⋯O2^vi^	0.90	1.91	2.810 (5)	178
N1—H1*C*⋯O5	0.90	1.86	2.761 (5)	177

Symmetry codes: (i) 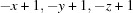; (ii) 
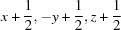
; (iii) 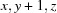; (iv) 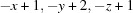; (v) 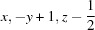; (vi) 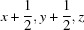.

**Fig. 1.**
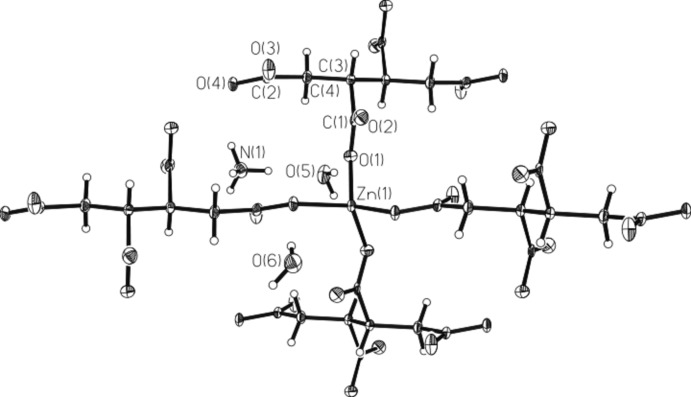
The molecular components of the structure of (I), showing the atom-numbering scheme and displacement ellipsoids at the 30% probability level.

**Fig. 2.**
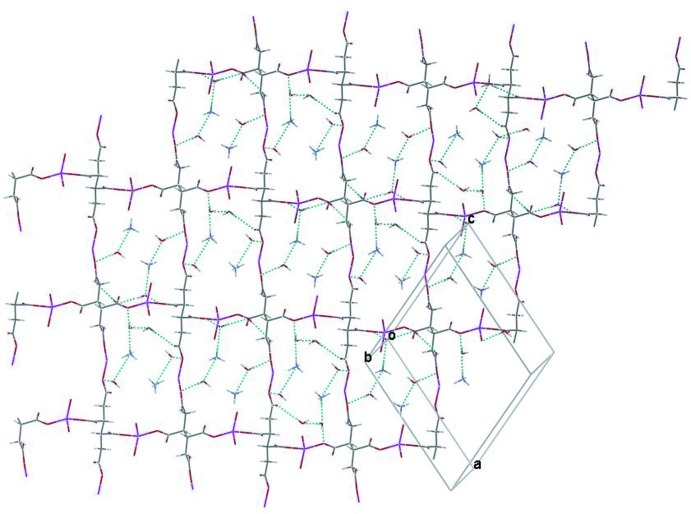
The packing of the structure of (I) showing the channel formation. Hydrogen bonds are displayed with dashed lines.

## supplementary crystallographic information

### Comment

Various derivatives of carboxylic acids have been widely used in pharmaceutical chemistry (Strachan *et al.*, 2007), supramolecular chemistry (Desiraju, 2002), and coordination chemistry (Rueff *et al.*, 2001). As an extension of our studies concentrating on coordination compounds with carboxylate ligands (Jin & Chen, 2007**a*,b*; Jin *et al.*, 2007), we report here the crystal structure of (NH_4_)_2_[Zn(C_8_H_6_O_8_)]^.^4H_2_O, (I).

The asymmetric unit of compound (I) contains half of the butane-1,2,3,4-tetracarboxylate anion, one ammonium cation, two water molecules and one Zn^2+^ cation. The butane-1,2,3,4-tetracarboxylate anion has an inversion centre located at the mid point of the central 3-C, and 4-C bond (symmetry code -*x*, -*y*, -*z*). The Zn^2+^ ion lies on a twofold rotation axis. It is surrounded by four O atoms from four symmetry-related butane-1,2,3,4-tetracarboxylate anions, forming a tetrahedral coordination geometry. Of the four coordinating O atoms, two come from the carboxylate groups in 1-position, while the other two come from the carboxylate groups in 3-position. The Zn—O bond lengths are almost equal.

The Zn^2+^ cations and the coordinating butane-1,2,3,4-tetracarboxylate anions form a three-dimensional network with channels extending along [110] and [010] (Fig. 2). In the channels water molecules and ammonium cations are present. They are hydrogen bonded to each other through O—H···O and N—H···O interactions and also hydrogen-bonded to the uncoordinating O atoms of the carboxylate groups of the anion.

Single crystals of the title compound were obtained by reacting zinc(II) acetate dihydrate with butane-1,2,3,4-tetracarboxylic acid in basic solution in the presence of 3,5-dimethyl pyrazole. However, 3,5-dimethyl pyrazole does not appear in the title compound. It should be noted that single crystals could not be obtained by evaporating an appropriate solution of the title compound in water or organic solvents. We found that it can be dissolved in a concentrated solution of ammonia; thus its single crystals were grown by slow evaporating its ammonia solution.

### Experimental

Butane-1,2,3,4-tetracarboxylic acid (22.3.1 mg, 0.10 mmol) was dissolved in 10 ml of methanol, to this solution zinc acetate dihydrate (42.6 mg, 0.2 mmol), and 3,5-dimethylpyrazole (19.2 mg, 0.2 mmol) was added. The solution was stirred for about 2 h at room temperature, and a large amount of precipitate formed. To the suspensition concentrated ammonia solution was added until the precipitate dissolved completely. The solution was filtered into a test tube and was left standing at room temperature. Several days later colorless block crystals could be obtained.

### Refinement

H atoms bonded to O, and N atoms were located in a difference Fourier map. Their bond lengths were constrained to values of O—H of 0.85 Å and N—H of 0.90 Å and they were allowed to ride on their parent atoms. All other H atoms were positioned geometrically with C—H = 0.97–0.98 Å, and constrained to ride on their parent atoms with *U*_iso_(H) = 1.2Ueq(C).

### Crystal data

**Table:**

(NH_4_)_2_[Zn(C_8_H_6_O_8_)]·4H_2_O	*F*(000) = 840
*M**_r_* = 403.65	*D*_x_ = 1.703 Mg m^−^^3^
Monoclinic, *C*2/*c*	Mo *K*α radiation, λ = 0.71073 Å
Hall symbol: -C 2yc	Cell parameters from 2092 reflections
*a* = 14.1153 (12) Å	θ = 2.8–27.6°
*b* = 8.8505 (8) Å	µ = 1.63 mm^−^^1^
*c* = 13.5704 (11) Å	*T* = 298 K
β = 111.761 (2)°	Block, colorless
*V* = 1574.5 (2) Å^3^	0.36 × 0.19 × 0.12 mm
*Z* = 4	

### Data collection

**Table:**

Bruker SMART 1K CCD area-detector diffractometer	1386 independent reflections
Radiation source: fine-focus sealed tube	1167 reflections with *I* > 2σ(*I*)
Graphite monochromator	*R*_int_ = 0.089
phi and ω scans	θ_max_ = 25.0°, θ_min_ = 2.8°
Absorption correction: multi-scan (*SADABS*; Bruker, 2002)	*h* = −16→16
*T*_min_ = 0.697, *T*_max_ = 0.823	*k* = −9→10
3841 measured reflections	*l* = −16→12

### Refinement

**Table:**

Refinement on *F*^2^	Primary atom site location: structure-invariant direct methods
Least-squares matrix: full	Secondary atom site location: difference Fourier map
*R*[*F*^2^ > 2σ(*F*^2^)] = 0.059	Hydrogen site location: inferred from neighbouring sites
*wR*(*F*^2^) = 0.156	H-atom parameters constrained
*S* = 1.02	*w* = 1/[σ^2^(*F*_o_^2^) + (0.1135*P*)^2^] where *P* = (*F*_o_^2^ + 2*F*_c_^2^)/3
1386 reflections	(Δ/σ)_max_ < 0.001
105 parameters	Δρ_max_ = 1.53 e Å^−^^3^
0 restraints	Δρ_min_ = −1.30 e Å^−^^3^

### Special details

**Table:**

Geometry. All e.s.d.'s (except the e.s.d. in the dihedral angle between two l.s. planes) are estimated using the full covariance matrix. The cell e.s.d.'s are taken into account individually in the estimation of e.s.d.'s in distances, angles and torsion angles; correlations between e.s.d.'s in cell parameters are only used when they are defined by crystal symmetry. An approximate (isotropic) treatment of cell e.s.d.'s is used for estimating e.s.d.'s involving l.s. planes.
Refinement. Refinement of *F*^2^ against ALL reflections. The weighted *R*-factor *wR* and goodness of fit *S* are based on *F*^2^, conventional *R*-factors *R* are based on *F*, with *F* set to zero for negative *F*^2^. The threshold expression of *F*^2^ > σ(*F*^2^) is used only for calculating *R*-factors(gt) *etc*. and is not relevant to the choice of reflections for refinement. *R*-factors based on *F*^2^ are statistically about twice as large as those based on *F*, and *R*- factors based on ALL data will be even larger.

### Fractional atomic coordinates and isotropic or equivalent isotropic displacement parameters (Å^2^)

**Table:**

	*x*	*y*	*z*	*U*_iso_*/*U*_eq_	
Zn1	0.5000	0.50029 (6)	0.7500	0.0196 (3)	
N1	0.6212 (3)	0.8163 (4)	0.5030 (3)	0.0379 (9)	
H1C	0.5861	0.8480	0.5427	0.045*	
H1D	0.6836	0.8588	0.5287	0.045*	
H1A	0.5884	0.8440	0.4350	0.045*	
H1B	0.6269	0.7150	0.5062	0.045*	
O1	0.41853 (19)	0.6486 (3)	0.6404 (2)	0.0291 (7)	
O2	0.3143 (2)	0.4535 (4)	0.5878 (2)	0.0340 (7)	
O3	0.3480 (3)	0.4657 (4)	0.3648 (3)	0.0413 (8)	
O4	0.44049 (18)	0.6425 (3)	0.32532 (19)	0.0262 (6)	
O5	0.5186 (2)	0.9191 (4)	0.6280 (3)	0.0516 (9)	
H5C	0.4923	0.8395	0.6420	0.062*	
H5D	0.5526	0.9635	0.6859	0.062*	
O6	0.6370 (2)	0.0991 (3)	0.7995 (2)	0.0470 (8)	
H6C	0.7008	0.0871	0.8163	0.056*	
H6D	0.6189	0.1787	0.7622	0.056*	
C1	0.3369 (3)	0.5846 (4)	0.5744 (3)	0.0210 (8)	
C2	0.3742 (3)	0.5970 (5)	0.3645 (3)	0.0244 (9)	
C3	0.2700 (3)	0.6794 (4)	0.4801 (3)	0.0203 (8)	
H3	0.2111	0.6180	0.4378	0.024*	
C4	0.3286 (3)	0.7247 (4)	0.4090 (3)	0.0254 (9)	
H4A	0.2827	0.7823	0.3496	0.030*	
H4B	0.3836	0.7918	0.4494	0.030*	

### Atomic displacement parameters (Å^2^)

**Table:**

	*U*^11^	*U*^22^	*U*^33^	*U*^12^	*U*^13^	*U*^23^
Zn1	0.0194 (4)	0.0239 (5)	0.0197 (5)	0.000	0.0122 (3)	0.000
N1	0.039 (2)	0.039 (2)	0.042 (2)	−0.0020 (16)	0.0213 (18)	−0.0064 (16)
O1	0.0260 (14)	0.0341 (16)	0.0247 (15)	0.0003 (12)	0.0064 (12)	0.0014 (12)
O2	0.0367 (17)	0.0293 (16)	0.0373 (18)	0.0002 (14)	0.0152 (14)	0.0075 (14)
O3	0.053 (2)	0.0317 (17)	0.056 (2)	−0.0036 (16)	0.0396 (18)	−0.0033 (15)
O4	0.0259 (13)	0.0337 (15)	0.0268 (14)	0.0008 (12)	0.0187 (12)	−0.0014 (11)
O5	0.061 (2)	0.055 (2)	0.053 (2)	−0.0072 (18)	0.0373 (18)	−0.0061 (17)
O6	0.0522 (18)	0.0453 (19)	0.0493 (19)	0.0042 (16)	0.0257 (16)	0.0129 (16)
C1	0.0232 (18)	0.028 (2)	0.0190 (18)	0.0074 (16)	0.0157 (16)	−0.0022 (15)
C2	0.0236 (18)	0.035 (2)	0.0190 (19)	0.0058 (17)	0.0126 (16)	−0.0008 (16)
C3	0.0197 (17)	0.026 (2)	0.0183 (18)	0.0006 (15)	0.0108 (15)	−0.0012 (14)
C4	0.0267 (19)	0.031 (2)	0.025 (2)	0.0040 (17)	0.0170 (17)	0.0035 (16)

### Geometric parameters (Å, º)

**Table:**

Zn1—O4^i^	1.996 (2)	O4—Zn1^i^	1.996 (2)
Zn1—O4^ii^	1.996 (2)	O5—H5C	0.8499
Zn1—O1^iii^	1.998 (3)	O5—H5D	0.8500
Zn1—O1	1.998 (3)	O6—H6C	0.8500
N1—H1C	0.9000	O6—H6D	0.8500
N1—H1D	0.9001	C1—C3	1.529 (5)
N1—H1A	0.9001	C2—C4	1.531 (5)
N1—H1B	0.9000	C3—C4	1.540 (5)
O1—C1	1.298 (4)	C3—C3^iv^	1.548 (7)
O2—C1	1.234 (5)	C3—H3	0.9800
O3—C2	1.220 (5)	C4—H4A	0.9700
O4—C2	1.300 (4)	C4—H4B	0.9700
			
O4^i^—Zn1—O4^ii^	101.42 (14)	O2—C1—C3	121.7 (3)
O4^i^—Zn1—O1^iii^	124.21 (10)	O1—C1—C3	116.9 (3)
O4^ii^—Zn1—O1^iii^	105.67 (10)	O3—C2—O4	124.1 (3)
O4^i^—Zn1—O1	105.67 (10)	O3—C2—C4	122.0 (4)
O4^ii^—Zn1—O1	124.21 (10)	O4—C2—C4	113.9 (3)
O1^iii^—Zn1—O1	97.83 (15)	C1—C3—C4	111.0 (3)
H1C—N1—H1D	108.3	C1—C3—C3^iv^	110.1 (3)
H1C—N1—H1A	109.9	C4—C3—C3^iv^	110.9 (4)
H1D—N1—H1A	109.9	C1—C3—H3	108.2
H1C—N1—H1B	109.9	C4—C3—H3	108.2
H1D—N1—H1B	109.9	C3^iv^—C3—H3	108.2
H1A—N1—H1B	108.9	C2—C4—C3	117.2 (3)
C1—O1—Zn1	110.2 (2)	C2—C4—H4A	108.0
C2—O4—Zn1^i^	121.1 (2)	C3—C4—H4A	108.0
H5C—O5—H5D	108.7	C2—C4—H4B	108.0
H6C—O6—H6D	108.6	C3—C4—H4B	108.0
O2—C1—O1	121.4 (3)	H4A—C4—H4B	107.2
			
O4^i^—Zn1—O1—C1	71.4 (2)	O1—C1—C3—C4	62.1 (4)
O4^ii^—Zn1—O1—C1	−44.7 (3)	O2—C1—C3—C3^iv^	118.4 (4)
O1^iii^—Zn1—O1—C1	−159.7 (3)	O1—C1—C3—C3^iv^	−61.2 (4)
Zn1—O1—C1—O2	5.1 (4)	O3—C2—C4—C3	15.6 (5)
Zn1—O1—C1—C3	−175.4 (2)	O4—C2—C4—C3	−165.7 (3)
Zn1^i^—O4—C2—O3	4.7 (5)	C1—C3—C4—C2	56.4 (4)
Zn1^i^—O4—C2—C4	−173.9 (2)	C3^iv^—C3—C4—C2	179.2 (3)
O2—C1—C3—C4	−118.4 (4)		

Symmetry codes: (i) −*x*+1, −*y*+1, −*z*+1; (ii) *x*, −*y*+1, *z*+1/2; (iii) −*x*+1, *y*, −*z*+3/2; (iv) −*x*+1/2, −*y*+3/2, −*z*+1.

### Hydrogen-bond geometry (Å, º)

**Table:**

*D*—H···*A*	*D*—H	H···*A*	*D*···*A*	*D*—H···*A*
O6—H6*D*···O4^i^	0.85	1.97	2.814 (4)	173
O6—H6*C*···O3^v^	0.85	1.99	2.833 (5)	173
O5—H5*D*···O6^vi^	0.85	1.97	2.805 (5)	168
O5—H5*C*···O1	0.85	1.98	2.816 (4)	167
N1—H1*B*···O2^i^	0.90	2.31	2.981 (5)	131
N1—H1*B*···O3^i^	0.90	2.30	3.009 (5)	136
N1—H1*A*···O5^vii^	0.90	2.54	3.153 (5)	126
N1—H1*A*···O6^viii^	0.90	2.24	2.948 (5)	135
N1—H1*D*···O2^ix^	0.90	1.91	2.810 (5)	178
N1—H1*C*···O5	0.90	1.86	2.761 (5)	177

Symmetry codes: (i) −*x*+1, −*y*+1, −*z*+1; (v) *x*+1/2, −*y*+1/2, *z*+1/2; (vi) *x*, *y*+1, *z*; (vii) −*x*+1, −*y*+2, −*z*+1; (viii) *x*, −*y*+1, *z*−1/2; (ix) *x*+1/2, *y*+1/2, *z*.

## References

[bb1] Bruker (2002). *SADABS*, *SMART* and *SAINT* Bruker AXS Inc., Madison, Wisconsin, USA.

[bb2] Desiraju, G. R. (2002). *Acc. Chem. Res.* **35**, 565–573. 10.1021/ar010054t 12118996

[bb3] Jin, S. W. & Chen, W. Z. (2007*a*). *Polyhedron*, **26**, 3074–3084.

[bb4] Jin, S. W. & Chen, W. Z. (2007*b*). *Inorg. Chim. Acta*, **12**, 3756–3764.

[bb5] Jin, S. W., Wang, D. Q. & Chen, W. Z. (2007). *Inorg. Chem. Commun.* **10**, 685–689.

[bb6] Rueff, J. M., Masciocchi, N., Rabu, P., Sironi, A. & Skoulios, A. (2001). *Eur. J. Inorg. Chem.* pp. 2843–2848.

[bb7] Sheldrick, G. M. (2008). *Acta Cryst.* A**64**, 112–122. 10.1107/S0108767307043930 18156677

[bb8] Strachan, C. J., Rades, T. & Gordon, K. C. (2007). *J. Pharm. Pharmacol.* **59**, 261–269. 10.1211/jpp.59.2.0012 17270079

[bb9] Westrip, S. P. (2010). *J. Appl. Cryst.* **43**, 920–925.

